# Respiratory Failure in an Extremely Premature Neonate with COVID-19

**DOI:** 10.3390/children8060477

**Published:** 2021-06-04

**Authors:** Vasantha H. S. Kumar, Arun Prasath, Clariss Blanco, Patrick O. Kenney, Christina M. Ostwald, Tracy S. Meyer, Cara F. Clementi, Richard Maciejewski, Mark T. Wilby, Anne Marie Reynolds, N Ja Hpa, Karl O. A. Yu

**Affiliations:** 1 Department of Pediatrics, Jacobs School of Medicine and Biomedical Sciences, University at Buffalo, State University of New York, Buffalo, NY 14203, USA; aprasath@buffalo.edu (A.P.); clarissl@buffalo.edu (C.B.); pokenney@buffalo.edu (P.O.K.); areynolds@upa.chob.edu (A.M.R.); njahpa@buffalo.edu (N.J.H.); kyu@upa.chob.edu (K.O.A.Y.); 2 Department of Medicine, Jacobs School of Medicine and Biomedical Sciences, University at Buffalo, State University of New York, Buffalo, NY 14203, USA; 3 Division of Infection Prevention, John R. Oishei Children’s Hospital, Buffalo, NY 14203, USA; COstwald@kaleidahealth.org (C.M.O.); TMeyer@kaleidahealth.org (T.S.M.); 4 Department of Pathology and Laboratory Medicine, Kaleida Health, Buffalo, NY 14203, USA; CClementi@kaleidahealth.org (C.F.C.); RMaciejewski@kaleidahealth.org (R.M.); MWilby@kaleidahealth.org (M.T.W.)

**Keywords:** COVID-19, SARS-CoV-2, respiratory failure, prematurity, infant

## Abstract

Coronavirus disease 2019 (COVID-19), a condition associated with SARS-CoV-2, typically results in mild infection in infants and children. However, children with risk factors such as chronic lung disease and immunosuppression have higher risk of severe illness from COVID-19. We report a case of a 27-week-gestation extremely premature infant born to a mother with COVID-19 infection. The infant, initially treated for surfactant deficiency, developed worsening hypoxic respiratory failure on the fifth day of life requiring escalating ventilatory support, an elevated level of C-reactive protein, thrombocytopenia, and an elevated level of d-dimer. The infant was positive for SARS-CoV-2 by RT-PCR from Day 1 to Day 42 of his life. The infant responded to a seven-day course of dexamethasone with a gradually decreasing oxygen requirement and could be extubated to non-invasive ventilation by the end of the fifth week after birth. The infant is currently on home oxygen by nasal cannula. Prolonged shedding of the virus may be a unique feature of the disease in premature infants. Extreme prematurity, immature lungs, and an immunocompromised status may predispose these infants to severe respiratory failure and a prolonged clinical course. Instituting appropriate COVID-19 protocols to prevent the spread of the disease in the neonatal intensive care unit (NICU) is of utmost importance. Infection with SARS-CoV-2 may have implications in the management of extremely premature infants in the NICU.

## 1. Introduction

The coronavirus disease 2019 (COVID-19) pandemic, caused by severe acute respiratory syndrome coronavirus-2 (SARS-CoV-2), has affected all aspects of human life and has created an extraordinary burden on global healthcare over the last year [1]. Despite the enormous amount of literature since the beginning of the pandemic in the adult population, information on the evolution of COVID-19 in pregnancy and newborn infants, especially in extremely premature neonates, is limited. COVID-19 infection, particularly pneumonia, has been reported in pregnant women [2]. However, no evidence of intrauterine infection of the fetus has been reported until recently. Several studies have suggested that vertical transmission is possible from mother to infant, based on IgM antibodies [3,4] and SARS-CoV-2 viral RNA detected from blood and nasopharyngeal (NP) swabs [4,5,6]. Neonatal infection with SARS-CoV-2 has been reported in late preterm infants following severe COVID-19 infection in the mother [5,7,8]. COVID-19 infection in extremely premature infants, notably, <28 weeks of gestation, is not reported, as most of the infections are reported late in pregnancy. Here, we report a case of respiratory failure due to perinatal transmission of SARS-CoV-2 infection in an extremely premature infant at 27-weeks of gestation, compromised by prematurity and respiratory distress syndrome with surfactant deficiency. Informed consent was obtained from the parents for the publication of the case report on their infant.

## 2. Case Report

A 27 2/7-week gestational age (GA) infant was born to a 32-year-old G5P4L4 mother, negative for HIV, HBsAg, and syphilis with a history of obesity and chronic hypertension. The mother was seen a day before delivery for abdominal pain and diarrhea, tested positive for SARS-CoV-2 by real-time PCR from a nasopharyngeal (NP) swab, was managed conservatively, and discharged home. She was admitted with profuse bleeding per vagina from a suspected placental abruption. The mother was administered betamethasone, magnesium sulfate, and antibiotics on admission for active labor. Chest X-ray (CXR) demonstrated patchy bilateral opacities, suspicious for COVID-19 pneumonia, requiring a 5 L/min flow of 100% O_2_ to maintain normoxia. Cesarean section was planned under spinal anesthesia for a frank breech presentation; however, the baby was delivered vaginally as the baby’s foot was noted at the introitus. A 27-week-gestational age (GA) male infant was delivered and transferred to the warmer for stabilization. It is to be noted that the baby was placed at least 6 feet away from the mother, with no direct contact at birth because of the mother’s positivity for SARS-CoV-2. The infant received positive-pressure ventilation (PPV) by the Neopuff (Fisher & Paykel Healthcare Ltd, Auckland, New Zeland) device for apnea and bradycardia. The infant was intubated for desaturations at 7 min of age, which resulted in a rapid increase in heart rate and SpO_2,_ and was able to wean to 0.21 FiO_2_ by 9 min of age. The APGAR scores were 1, 5, and 7 at 1, 5, and 10 min, respectively, and he was transferred to the neonatal intensive care unit (NICU) for further management. The infant was appropriate for gestational age, with a birth weight of 1185 g (79th percentile on Fenton curves) [9].

**Figure 1:**
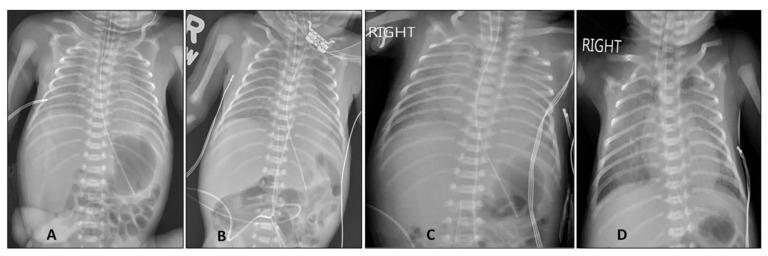
Progression of chest X-ray in the first two weeks after birth. (**A**). Chest X-ray soon after birth demonstrated low lung volumes and air bronchograms from hyaline membrane disease. (**B**). Chest X-ray at 48 h of age demonstrated better lung expansion after three doses of intratracheal surfactant administration. (**C**). The infant was in hypoxic respiratory failure, requiring 100%O_2_ on day 5 after birth. Chest X-ray revealed significant bilateral opacities with low lung volumes. (**D**). A dexamethasone course significantly improved lung aeration, with decreasing lung opacities on day 12 after birth.

On admission to the neonatal unit, the infant was administered three doses of surfactant (Infasurf, ONY Corporation, East Amherst, NY, USA) in the first 48 h of admission for a chest X-ray typical of hyaline membrane disease (Figure 1A,B) and an increasing FiO_2_ (0.3–0.4). The infant’s mechanical ventilation post-surfactant was stable on a FiO_2_ of 0.3 and tidal volume of 4.5 mL/kg. The infant was cared for with appropriate personal protective equipment (PPE), including N95 masks and established NICU COVID-19 protocols, pending RT-PCR results. 

The infant’s respiratory status worsened on day 5, with the development of severe hypoxic respiratory failure (HRF) as indicated by extensive opacities on chest X-ray (Figure 1C), requiring 100% O_2_ and high-frequency oscillatory ventilation (HFOV). Of note, the infant had an elevated level of C-reactive protein (31.7 mg/L), thrombocytopenia, and a moderately elevated level of D-dimer (1.17–1.34 µg/mL), along with a worsening clinical status. The differential diagnosis included bacterial or fungal infection, pneumonia from *Ureaplasma* or *Mycoplasma hominis*, other viral etiologies including SARS-CoV-2. Nasopharyngeal swabs from the infant tested positive for SARS-CoV-2 virus by RT-PCR at 24 and 48 h of age (Table 1). In the presence of positive results for SARS-CoV-2, a worsening clinical status, and a negative sepsis work-up including a viral respiratory screen, SARS-CoV-2 pneumonia and potential systemic COVID-19 infection was considered.

Antibiotics were administered (ampicillin with cefotaxime) for suspected sepsis and worsening respiratory status for seven days. Azithromycin was administered for three days for possible *Ureaplasma* pneumonia (respiratory cultures were negative). The fourth dose of surfactant was administered on day 6 for persistent desaturations on FiO_2_ of 1.0 for HRF with good results. The infant remained on HFOV from day 5 to day 21 after birth.

Dexamethasone was administered at 0.15 mg/kg/day every 12 h for seven days on days 5 to 12, with significant improvement on chest X-ray (Figure 1D). The infant was started on daily inhaled budesonide (500 µg daily) on day 12. The infant was not a candidate for remdesivir due to his extreme prematurity and low birth weight. The infant responded to dexamethasone with a gradual fall in C-reactive protein levels and decreasing FiO_2_ requirements over 1–2 weeks and was switched to a conventional ventilator on day 21. The infant was extubated to non-invasive ventilation (NIV) on FiO_2_ of 0.3 on day 37 and weaned further to a high-flow nasal canula (HFNC) on day 55. The infant was discharged home on daily budesonide and home oxygen therapy (0.5 L 100%O_2_) via the nasal canula at 42 weeks post menstrual age (PMA).

The infant was periodically tested for SARS-CoV-2 both by NP swab and from the ET tube secretions (Table 1). Most tests were run with a two-target real-time PCR (*S* and *ORF1ab* genes, DiaSorin Molecular, Cypress, CA, USA). The infant continued to test positive for SARS-CoV-2 on days 2, 14, 22, 30, and 42 of age. Real-time PCR cycle threshold (Ct) values are inversely related to the amount of nucleic acid in a specimen sample. As such, while Ct values depend upon specimen collection factors and quality, Ct values can also provide an informal measure of both the patient’s infectivity and viral load. The patient had lower Ct values initially, suggesting higher viral loads close to birth. Over time, increasing Ct values indicated a gradually decreasing viral load, with negative tests reported on days 50 and 57 (Table 1). The parents visited their infant at resolution of their infection in full personal protective equipment (PPE) with airborne precautions until the time the infant was taken off precautions after the second negative test. 

A non-occlusive thrombus (4.2 × 2.5 × 3 mm) in the inferior vena cava (IVC) was noted on day 5 at the tip of the umbilical venous catheter (UVC). The catheter was removed, given that the thrombus was likely related to the presence of the UVC. The infant received one platelet transfusion, and his platelet count stabilized above 100,000/mm^3^ by day 13. Anticoagulation therapy was deferred during the initial management of the thrombus in view of the risk of a life-threatening hemorrhage in a critically sick premature infant. Serial doppler studies of the IVC demonstrated a gradual decrease in the size of the thrombus over time. The infant was treated with IV acetaminophen for five days for moderate to large ductus arteriosus in the third week after birth. A repeat echocardiogram post-treatment demonstrated a small ductus. Bilateral grade I intraventricular hemorrhage was noted on serial head ultrasounds, with no hydrocephalus. The infant is currently on ad lib feeds of Enfacare 27 Kcals/oz formula, with a weight of 4230 g (56th percentile on Fenton curves).

## 3. Discussion

We report a case of severe acute respiratory infection with hypoxic respiratory failure and likely COVID-19 pneumonia in a 27-week-GA extremely premature infant. The infant had problems associated with extreme prematurity, such as surfactant deficiency from hyaline membrane disease and a patent ductus arteriosus. The infant improved after surfactant administration and then worsened to develop HRF. The diagnosis of SARS-CoV-2 infection was considered because of a negative sepsis work-up, NP swab positive for SARS-CoV-2 virus, and presence of systemic signs of COVID-19 infection, such as an elevated level of C-reactive protein and thrombocytopenia in the infant. The clinical improvement with dexamethasone and a normalizing C-reactive protein level also strengthened the diagnosis of COVID-19. In adults, older age, male sex, and co-morbidities increase the risk of COVID-19 pneumonia and a prolonged clinical course [10]. Dexamethasone treatment improves mortality from severe and critical COVID-19 in adult patients [10]. An immature immune system and the lack of protective maternal antibodies due to extreme prematurity may have contributed to the prolonged shedding of SARS-CoV-2, resulting in severe HRF and a protracted course in the infant. The duration of invasive and non-invasive ventilation (55 days) is not unusual in an extremely premature infant. However, it is not typical for infants born with a weight >1000 g to be mechanically ventilated for prolonged periods. SARS-CoV-2 virus may have contributed to the prolonged ventilation and respiratory support in this infant. The infant was SARS-CoV-2-positive for a prolonged period of six weeks, with Ct values not reaching the range wherein culturable virus can be confidently ruled out (e.g., >34 cycles) [11]. COVID-19 has been reported in an extremely premature infant positive for SARS-CoV-2 virus by the end of first week after birth [12]. However, unlike our case, the infant had no clinical or biochemical signs suggestive of COVID-19 infection. There is paucity of literature on the correlation between prolonged shedding of SARS-CoV-2 and inhaled budesonide administered to treat chronic lung disease of prematurity. However, early administration of inhaled budesonide has been shown to reduce the need for urgent medical care and recovery time from COVID-19 in adults in a randomized control trial [13]. 

Thrombotic occlusion of the central venous catheters such as UVC are commonly seen in premature neonates [14]. Sick, premature infants are particularly prone to thrombus formation [15], particularly in the presence of pro-inflammatory states such as systemic COVID-19 infection [16]. A low platelet count and elevated C-reactive protein and D-dimer levels favoring a hypercoagulable state may be possible contributing factors in the formation of the thrombus. 

The mother was positive for SARS-CoV-2 infection, and the infant was positive for SARS-CoV-2 by 24 h of age, indicating the possibility of horizontal transmission at delivery, although vertical transmission of the infection cannot be ruled out. However, defining vertical transmission based on isolated case reports is difficult. Antibody titers could have helped in differentiating vertical from horizontal transmission. However, the absence of maternal and infant antibody titers and placental cultures are the limitations of the report. Several studies have suggested that vertical transmission is possible from mother to infant, based on IgM antibodies [3,4] and SARS-CoV-2 viral RNA isolated from blood and nasopharyngeal swabs [4,6]. Transplacental transfer of SARS-CoV-2 in a neonate born to a mother who became infected with the virus in the third trimester has been reported [17]. A systematic review of 11 studies did not demonstrate any intrauterine or transplacental transmission of the SARS-CoV-2 virus to the fetus during the third trimester of pregnancy [18]. In a large study of 101 newborns born to Covid-19-positive mothers, no evidence of perinatal transmission was observed, despite two infants having results demonstrating low viral loads [19]. Current evidence suggests that the likelihood of perinatal transmission of SARS-CoV-2 is very low [20]. Despite the weight of the evidence demonstrating the lack of in utero transmission of SARS-CoV-2, the expression of angiotensin-converting enzyme 2 (ACE2), the receptor for SARS-CoV-2, on maternal–fetal interface cells such as stromal cells, cytotrophoblasts, and syncytiotrophoblasts on the placenta and other fetal organs [21] may suggest that vertical transmission is possible and needs to be investigated further.

COVID-19 infection in extremely premature infants should be taken seriously from an infection control standpoint, with appropriate PPE measures to prevent the spread of the infection in the NICU. Minimizing aerosol generation in ventilated patients and during extubation is likely needed to prevent secondary infection of healthcare workers, visitors, and other patients. The most recent guidance by the AAP focuses on using infection control measures to support maternal–newborn contact and breastfeeding [22] to prevent infection in the newborn. 

In conclusion, we present a case of severe respiratory failure from SARS-CoV-2 infection in an extremely premature infant with prolonged viral test positivity. Infection control measures during delivery, maternal–infant contact, breastfeeding, and postnatal care of the infant are essential to minimize the risk of transmission, both to and from the infant and the healthcare team. 

## Figures and Tables

**Table 1:** SARS-CoV-2 test results and viral quantification by cycle threshold (Ct) values by real-time PCR over time.

Day after Birth	Specimen	SARS-CoV-2 Result	Ct Value (S Gene)	Ct Value (ORF1ab Gene)
Day 1 (24 h)	Nasopharyngeal	Positive	17	17
Day 2 (48 h) *	Nasopharyngeal	Positive		
Day 14	Nasopharyngeal	Positive	27	27
Day 22	Nasopharyngeal	Positive	31	30
Day 30	Nasopharyngeal	Positive	31	33
Day 30	Endotracheal	Positive	33	ND
Day 42	Nasopharyngeal	Positive	28	28
Day 50	Nasopharyngeal	Negative	ND	ND
Day 57	Nasopharyngeal	Negative	ND	ND

SARS-CoV-2 real-time PCR testing was performed using an FDA emergency use authorization kit for Liaison MDX (DiaSorin Molecular, Cypress, CA, USA), except for the day 2 specimen (*), for which it was performed with the Panther System (Hologic, San Diego, CA, USA). With the Panther system, PCR was done using two targets in the *ORF1ab* gene, and no quantitation is reported. Ct–cycle threshold; ND–not detected.

## Data Availability

Not applicable.
